# Exploring Perceived Importance of a Novel Emergency Food Program during COVID-19 and Program Recipient Characteristics

**DOI:** 10.3390/ijerph182010786

**Published:** 2021-10-14

**Authors:** Makenzie L. Barr, Kendra OoNorasak, Kristin Hughes, Lauren Batey, Kaela Jackson, Haley Marshall, Tammy Stephenson

**Affiliations:** 1 Department of Dietetics and Human Nutrition, University of Kentucky, Lexington, KY 40506, USA; kendra.oonorasak@uky.edu (K.O.); lauren.batey@uky.edu (L.B.); kaela.jackson@uky.edu (K.J.); tammy.stephenson@uky.edu (T.S.); 2 FoodChain Nourish Lexington, Lexington, KY 40508, USA; kristin@foodchainlex.org; 3 Department of Internal Medicine and Pediatrics, University of Kentucky, Lexington, KY 40506, USA; hnma239@luky.onmicrosoft.com

**Keywords:** COVID-19, food access, community, food security

## Abstract

Following rising unemployment rates and consequent loss of income due to COVID-19, many people have been seeking meal assistance. This study examines the impact of a community-based free meal distribution program during the pandemic in Kentucky, reviewing characteristics of recipients of the program. Demographics, health behaviors, food insecure classification, and rating of importance of the meal program were collected. Qualitative feedback on the impact of the program was collected via open response. Of the 92 participants using the meal service, the cohort was female, Black, 43 years of age (43.5 ± 15.0 years), with a household income under 30,000 USD before COVID, decreased income since COVID, and were food insecure. Recipients rated the importance of the service as 8.7 ± 1.8 (of 10), and those with children indicated the importance as 4.2 ± 1.1 (of 5). Qualitative data on program importance highlighted four response categories including “changed habits”, “mental wellbeing”, “provided resources”, and “other”. In response to the COVID-19 pandemic, many individuals have struggled. Meal assistance programs are a fundamental asset in the community that have seen marketed demand since COVID-19. Collaboration with, and evaluation of, meal assistance programs can be valuable for continued programmatic funding support.

## 1. Introduction

The Coronavirus Disease (COVID-19) pandemic has led to devastating impacts on the financial security, health, and well-being of millions of individuals and families across the United States. According to the Bureau of Labor Statistics, in the spring of 2020, 47 of 50 states reported historically high unemployment rates, surpassing levels experienced during the Great Depression [1]. The Center for Community Solutions found that two thirds of residents living below the poverty line had struggled to pay for at least one necessity in the past year including food, gas, phone, and utility bills [2]. These problems were exacerbated during the early months of the pandemic when more than 40 million unemployment claims were filed, and a survey from the Social Policy Institute at Washington University reported that 24% of respondents had lost a job or income due to COVID-19 [3]. Consequently, within the first month of the pandemic, about 31% of adults in the U.S. reported that they could not pay rent, mortgage, or utility bills, or were forced to go without medical care [4].

As a result of the pandemic and subsequent economic downturn, American families are also facing additional risks resulting from increased food insecurity and reduced access to nutritious meals. Food insecurity (FI) refers to individuals or households that at some point during a given year had difficulty obtaining enough food due to a lack of resources [5]. The United States Department of Agriculture (USDA) defines FI as a lack of access, at any point, to enough food for an active, healthy life for all household members and limited or uncertain availability of nutritionally adequate foods. FI status is determined by standardized, validated USDA food security survey modules that vary in length from 6–18 questions [6]; however, the utilization of a two-item food security survey tool has been validated for families [7]. Comprehensive research conducted in 2018 for the Map the Meal Gap project estimated that there are more than 37 million food insecure individuals in the United States [8]. Frequency of FI will increase throughout the COVID-19 pandemic as economic impacts continue. It was projected that FI would increase by 17 million more Americans [9]. One study reported an increase of 20% in households reporting FI following the economic downturn [10]. In early 2020, a survey found that about 30.6% of adults reduced their families’ spending on food following the initial shutdowns, and that that number increased to 46.5% for families that lost work or income [4]. Following this reduction in food spending, studies have shown a dramatic increase in FI compared to previous years. It was reported in 2018 that the percentage of households that were food insecure was about 13%, and that percentage increased to about 15% for households with mothers with children under the age of 12 [11]. By the end of April 2020, more than one in five households in the US, and two in five households with mothers with children under the age of 12, were food insecure [11].

Research has shown that FI leads to negative consequences regarding diet quality and nutritional status. It may also lead to a change in family eating habits and the home food environment. Often, high calorie processed foods are more affordable than healthier alternatives, leading to an increase in poor nutritional consumption for families and children. Six months into the pandemic, respondents to one survey indicated a 47% increase in the purchase of high calorie, nonperishable, processed foods, with about one third of participants reporting an increase in the consumption of snack foods, desserts, and sweets [10]. FI is also linked with an increased likelihood of poor dietary patterns including lower intake of fruit, vegetables, and fiber, and an increased intake of energy dense foods [12]. Thus, because of this impaired access to, and inadequate intake of nutritious food, FI is closely associated with an increased risk of chronic disease such as being overweight, obesity, type 2 diabetes, hypertension, and ultimately, higher healthcare costs [13,14,15,16,17,18]. The negative impacts of FI extend beyond diet quality, nutritional status, and diet-related diseases, as FI is associated with poor sleep outcomes, oral health problems, mental health problems including depression, and poor health [19].

As the pandemic continues and the length of closures remains uncertain, the country has entered uncharted territory regarding food resource allocation. Food banks and soup kitchens have seen a marked increase in the need for their services [8]. Feeding America reports that about 4 out of every 10 visitors to food banks across the country are there for the first time, and from March through to October of 2020, food banks distributed an estimated 5 billion pounds of food, which is the equivalent of 4.2 billion meals nationally [20,21]. Between May 2020 and May 2021, the USDA worked to tackle local food systems and FI issues through their Farmers to Families Food Box Program which provided fresh food to families in need with the coordinated assistance of local food banks and food distributors. The Farmers to Families Food Box Program has been a fundamental resource, distributing more than 173 million boxes nationwide [22].

Despite these efforts, southern and rural areas have particularly struggled during COVID-19, as FI is additionally impacted by a lower average socioeconomic status [13,14]. This is particularly concerning for the top 10% of counties with the highest levels of FI where 78% are rural and 87% are located in the South [8]. An example of heavily impacted state would be Kentucky (KY), a largely rural state, due to a highly prevalent FI rate of 14.7% which is well above the national average of 11.1% before the pandemic [5]. There are federal and/or state food assistance programs, including Supplemental Nutrition Assistance Program (SNAP) benefits for individuals and families who have a gross income below 130 percent of the federal poverty level in the U.S. (e.g., less than 16,744 USD annual gross income for a one-person household and less than 34,450 USD yearly gross income for a family of four in Kentucky) [23]; however, FI status is not determined by the income level and those who are experiencing some level of food insecurity may not be eligible for many assistance programs that determine eligibility based on income only. In an urban city of central KY, emergency food programs have been working to support the community and combat the dramatic increase in the demand for nutritious food. While some food pantries and soup kitchens require that individuals are low-income to be eligible for food assistance, some non-profits do not. One such organization expanded their reach without limiting recipients to only to low-income individuals through the employment of recently furloughed hospitality workers, prioritization of sourcing local foods to support the local economy, and contacting local restaurants to assist with meal preparation and help with paying employees. Furloughed workers from across the hospitality industry were recruited and worked in shifts to help prepare meals and distribute them throughout the community. Local restaurants and caterers, who were closed to the public during the shutdown, prepared and distributed meals to some of the community sites. To support local schools with meals for kids, this organization, through the assistance of several restaurants and caterers, provided meals at several community sites throughout the city, making meals accessible to all students in the district. These efforts ensured the community was being nourished and workers and restaurants were still earning a wage. As of May 2021, they have provided at least 302,000 meals to 62 sites in the local community since the beginning of the pandemic, with over 1,000,000 USD invested into the local food economy and 35,000 pounds of local produce purchased [24,25,26].

As we move forward in the COVID-19 pandemic, understanding the need for and importance of preventing FI has never been more important. Though emergency food assistance programs have responded to the increased needs and demands of their communities, research on the impact of these pandemic relief programs is still limited. This study aims to fill that gap in our understanding. The objectives of the current study are to (1) characterize the impact of the COVID-19 pandemic on health behaviors as well as personal and financial well-being, and (2) assess the impact of a community-based free meal distribution program providing emergency support during a pandemic. By researching the impact these programs may have on the lives and health of their recipients, we can provide detailed information for the development and funding of current and future emergency food programs.

## 2. Materials and Methods

Data were collected through an anonymous cross sectional, web-based survey [27] (Qualtrics survey platform version September 2021) of individuals utilizing free meal services in KY during the COVID-19 pandemic. Participants were recruited between September 2020 and February 2021.

As a result of the COVID-19 pandemic, FoodChain (one of the largest sites supplying meals in this study), a 2011 established sustainable food organization, expanded its typical efforts to employ displaced food workers, support local farms, and provide thousands of meals to those in need [24,25]. The program is one that supplied meal services throughout the county and to local schools during closures. Marketing for the program was shared through social media pages, press releases within the community, resource connections such as United Way and the city government website, as well as via word of mouth. Meal recipient participants were recruited face-to-face at meal distribution sites (FoodChain sites and at other community sites nearby) via 1385 meal distributed paper flyers, yard signs at the meal distribution sites while individuals waited in line for their meals, and through social media marketing on their Facebook page. Inclusion criteria for recipients of the meal assistance program included being over the age of 18 years, able to read and understand the English language, and having received a free meal at the time of data collection (September 2020 through February 2021). Individuals received a 20 USD gift card incentive for their time completing the survey.

### 2.1. Recipient Characteristics

Descriptive variables included sex, age, race, income level, job status (working for pay, income before COVID-19 and resulting income due to COVID-19). FI status calculated from the Hager two-item screener (“Within the past 12 months we worried whether our food would run out before we got money to buy more” and “Within the past 12 months the food we bought just didn’t last and we didn’t have money to get more”) [7]. Food insecurity was assessed if at least one of the two-item questions were affirmative (often or sometimes).

Health-related variables were captured to examined health disparities through seven survey items assessing if a doctor or healthcare provider has ever told them they had one of the following conditions: diagnosed type 2 diabetes mellitus, high cholesterol, hypertension, overweight or obesity, cancer, anxiety, or depression. Stress was additionally self-assessed for pre-COVID and post-COVID through the ten-item perceived stress scale (PSS-10; 0–40, increased stress with higher score). For pre-COVID stress, survey respondents were asked to recall how they felt or perceived before the COVID-19 pandemic regarding the ten statements of the PSS-10 scale. Finally, dietary intake was assessed from “In the last seven days, how many days did you eat 4.5 servings or more of fruit and vegetables per day (ex: one serving of fruit is a medium apple, one banana; one serving of vegetable is a cup of spinach, half of a large potato)?” and “which food groups are missing from your diet that you don’t eat everyday (grains, protein, vegetables, fruits, and dairy)?”.

### 2.2. Program Importance

Meal program importance and perceptions were collected. Ten Likert-item statements were asked on a scale from 1 (strongly disagree) through to 10 (strongly agree) to assess the program’s capacity to improve dietary habits, meet dietary preferences, and improve sense of belonging. Nine positive ranked items included the program (1) gave me a sense of belonging in the community, (2) introduced me to new food(s) that is/are not part of my diet, (3) decreased my level of stress in terms of food access, (4) met my dietary needs, (5) meals were visually appealing, (6) met my taste preferences, (7) were good quality, healthy meals, (8) had a positive impact on my ability to access fruits and vegetables, (9) had a positive impact on my eating habits. One negative Likert item detractor was used to ensure reduction of participant bias (“The program had a negative impact on my budgeting skills”).

Importance and convenience of the meal program to participants and their families were examined by four questions: three Likert item questions (“How essential is/was this free meal service to the food intake of your family during the COVID-19 pandemic?”, “How much does this program help food intake for your child or family during the COVID-19 pandemic?”, and “How convenient is/was it for you to access your meal distribution site each time that you needed it?”). Finally, one open ended qualitative item (“In what way has this meal service impacted your life since the pandemic began?”) was asked to allow for more detailed explanation from community members on the specific ways the meal service helped them during this time.

### 2.3. Statistical Analyses

Descriptive statistics are shown by frequencies and measures of central tendency (mean and standard deviation). Demographic variables were assessed by meal distribution recipient (indicated “yes” to using meal service in county).

Thematic analysis was used to examine qualitative feedback from one open-ended survey item asked of meal program recipients. Data were coded for major themes and sub-themes. Survey responses were coded by two trained independent researchers. The two initial independent researchers studied the open-ended responses line by line to make general notes. Notes were used to develop general categories. These categories were synthesized into main sub-themes and ultimately combined into overarching main themes. Themes were reviewed by a third, trained outside reviewer, for any discrepancies and reached 100% agreement of themes and sub-themes. Data are presented in frequency along with illustrative example quotes.

## 3. Results

### 3.1. Recipients Characteristics

The survey response rate was 6.6%. Of 92 participants who used the meal service, the cohort was predominantly female (71.7%), Black (52.7%), approximately 43 years of age (43.5 ± 15.0 years), had a household income under 30,000 USD before COVID-19 (52.2%), had decreased income as a result of the pandemic (45.6%), had increased unemployment since the COVID-19 pandemic (*n* = 14) and were classified as food insecure (94.5%) (Table 1).

The population in Fayette County where main meal distribution sites were located is relatively White (77.2%), has a median household income of 57,291 USD, and an unemployment rate of 4.7% (rates in September 2020 at beginning of data collection). Likewise, the state of Kentucky is also predominately White (87.5%), has a median household income of 50,589 USD, and an unemployment rate of 5.5% (September 2020, representative data for the time frame of study data collection).

Many individuals in the cohort responded to having been told by a doctor or healthcare professional that they had been diagnosed with a chronic health condition. Predominate conditions in the group were anxiety (53.0%), being classified as overweight (52.9%), high blood pressure (44.6%), and depression (43.4%). Additional conditions included classified obesity (34.9%), high cholesterol (34.1%), type II diabetes mellitus (22.9%), and cancer (11.0%). Since the pandemic, participants PSS-10 score was elevated (7.1 ± 1.6; 15.6 ± 5.3 pre-COVID vs. 22.7 ± 6.8 since), and stress significantly increased (mean difference = 8.15). Among missing food group items, recipients were predominately lacking in fruits (46.7%), vegetables (41.3%), and dairy (33.7%), followed by protein (27.2%) and grains (21.7%).

### 3.2. Program Importance

For meal assistance program recipients, over 71% were using meal assistance services more frequently since the COVID-19 pandemic, rated the importance of the surveyed program as 8.7 ± 1.8 (out of 10), and of those with children (*n* = 39) indicated importance as 4.2 ± 1.1 (out of 5). Of individuals who were utilizing these meal assistance programs (food pantry, soup kitchen, community center, etc.) more frequently since the start of the COVID-19 pandemic, they also rated the importance of the program significantly higher than those using meal programs the same amount or less since COVID-19 (*p* = 0.03). When asked on a Likert item of 1 (least convenient) to 10 (most convenient) to rank how convenient the meal distribution sites were to access recipients reported an average of 8.4 ± 2.1 (of 10).

**Figure 1:**
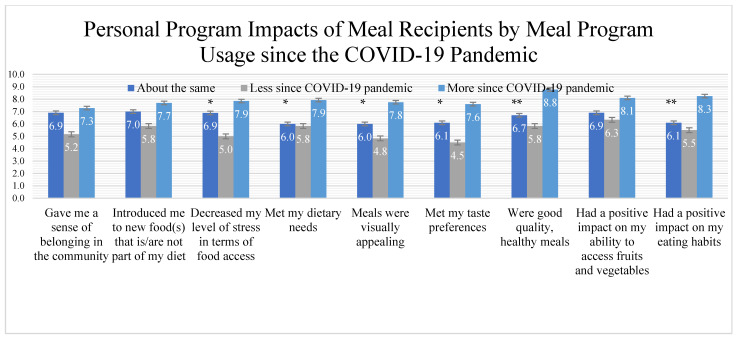
Likert Item Statements of Personal Impact of the Meal Service Program for Recipients. Data reported in means and standard error; * indicates *p* < 0.05 significant differences by utilization of meal services the since COVID-19 pandemic; ** indicates significance of *p* < 0.01.

To examine the program-specific feedback from meal program recipients, ten items were asked on a Likert scale from 1 being strongly disagree to 10 being strongly agree (Figure 1). One detractor statement was used to reduce participant response bias which was, the program “had a negative impact on my budgeting skills” in which the mean score was 3.83 out of ten (not shown). Among grouping of utilization of meal program services since the COVID-19 pandemic, 11 participants report using services “about the same” amount, 6 report using services “less since COVID-19”, and 41 report using services “more since the pandemic” (*n* = 58 completed all questions; data shown in Figure 1). For participants utilizing services the same amount or more, items were rated at a 6.0 or higher with “good quality, healthy meals” being the highest rated item. For those using meal assistance services less since COVID-19, all items were rated lower than comparative groups. On six of the Likert items, there were significant differences between service utilization grouping with those using meal assistance services more since the pandemic rating the importance of the program more positive (all *p* < 0.05). Likert items were examined by demographic variable groups (sex, race, age, and income level) with no significant differences (all *p* > 0.05).

Open-ended response data from one survey item (“In what way has this meal service impacted your life since the pandemic began?”) was completed by 79 participants. Four main qualitative themes were identified through summative analysis of responses. Main themes identified included (1) changed habits (e.g., healthier eating; *n* = 17), (2) mental wellbeing (e.g., reduced stress regarding food intake; *n* = 27), (3) provided resources (e.g., extra food, budgeting; *n* = 59), and (4) other (e.g., negative; *n* = 5). Main themes were categorized into sub-themes and listed in Table 2.

#### 3.2.1. Changed Habits

Of participants that described changed habits in relation to the meal service program, eating habits were altered among them and their families. Their meal program participation allowed them to have access to healthier food options that they may not have had access to without. Three participants additionally shared about the program changed their dietary intake such as by eating foods they were “unfamiliar with and enjoyed”, and that they were provided “delicious meals” they otherwise wouldn’t make at home, and as a result, ate “more veggies”.

#### 3.2.2. Mental Wellbeing

Our second largest theme concerned mental wellbeing during the COVID-19 pandemic in relation to the meal assistance service. One participant thoroughly stated that “FoodChain has kept my mental and emotional well-being intact due to not having to stress over meal planning and execution or worrying about my kids next meals.” Others highlighted that picking up meals from the distribution sites got them out of the house, boosted morale, or gave them the opportunity to hear words of encouragement.

#### 3.2.3. Provided Resources

As this meal service, in and of itself, was providing a resource to the community, this theme was the most frequently shared among participants. The service improved access, assisted with financial strains, and provided for family and/or community members. Shared sentiments that the program (1) kept meals on the table, (2) allowed participants to utilize money for other necessities (medication, household needs), and (3) reduced frequency of leaving the house.

#### 3.2.4. Other

A few comments were shared by participants that, although outside the general scope of comments, are important to highlight. General comments shared included that the program “helped me tremendously” and “it helped me in a big way which I am very thankful for”. Interestingly one participant shared that the program taught them to stretch their groceries for more meals while another participant stated their eating habits were worse.

## 4. Discussion

Though FI is an existing and prevalent issue that has been addressed by food assistance programs prior to the pandemic, there is limited evidence on the impact of emergency food assistance programs in the midst of the COVID-19 pandemic. In this study we aimed to describe the population of recipients being served at a local food assistance program during COVID-19 and understand the personal impacts this program made on their lives. The main findings support the importance of meal assistance programs to community members, particularly those with children or families. Of our meal program recipients, diagnosed health conditions were prevalent, fruit and vegetable serving intake was low, and self-perceived stress increased since COVID-19.

Of the current cohort, unemployment rates rose. Nationally, unemployment hit an all-time high in April 2020 and a study reported that households who experienced COVID-19 related job losses were, understandably, twice as likely to struggle financially in comparison to those who did not experience any job or income loss [3]. Resembling our cohort demographics, most participants from the study led by Despard and colleagues earned less than 30,000 USD [3]. While federal, state, and regional assistance has been provided to alleviate the short-term impacts of the pandemic, long-term economic consequences are far from over. A study from the Pew Research Center found that about one half of adults in low-income households have taken on debt to survive during the pandemic [28]. This debt means that even after returning to work, many will continue to struggle to afford necessities including food. Due to economic hardships related to COVID-19, consequent hardship upon families’ housing, nutrition, and food security, and health status has amplified. For millions of individuals, sustaining access to basic needs was more challenging, attracting the need for community and federal assistance.

Due to job loss, resulting FI, and life in the pandemic as a whole, stress is consequently amplified. Stress significantly increased from pre-COVID to post-COVID times for program recipients. A decline in mental health is not unique to the population we’ve studied; the prevalence of depression symptoms in American adults since the pandemic has increased on average [29]. For individuals with lower social and economic resources and added stressors such as job loss, depressive symptoms were reported more frequently [29]. Similar trends in increased depression among recipients was also recorded in our study. Continued support for meal assistance programming and food assistance benefits can aid in addressing worry, concern, or stress surrounding making ends meet or putting food on the table.

Food assistance programs that extend beyond the provision of food and/or meals with community empowerment, training, and education have the potential to not only address FI in the short-term, but also target the root causes of FI and improve social belonging of marginalized populations. Such programs could build community capacity, advocate FI issues through actions, emphasize the need to go beyond the food pantry model, and highlight impacts and possible investment-worthy models to policymakers. Evaluation of food assistance programs not only lies in understanding the effectiveness of the program but through consideration of the needs of, and impact on, the participants that use the program. Of our population, the recipients of this program were older in age, predominantly minority residents, in a low-income category, food insecure, and were not meeting the recommended 4.5 servings of fruits and vegetables each day. Adams et al. (2020) explains that more frequent purchases of highly processed foods and reduced purchases of fresh fruits and vegetables were notable among families, particularly those who were food insecure, since many families shopped at grocery stores less often to minimize social exposure during the pandemic [3]. Despard et al. (2020) further describes that one third of adults reported reducing money spent on food due to the pandemic [3]. On the other hand, the importance of produce was noted in a 2015 meta-analysis, stating that high fiber fruit and vegetables reduced the risk of developing type 2 diabetes, a chronic disease that is significantly more prevalent among meal recipients [30]. Low consumption of fresh produce among recipients may increase their likelihood of being diagnosed with nutrition-related medical conditions especially with more recipients having a nutrition-related diagnosis, though more research needs to be conducted to explore this potential relationship.

In addition to providing meal assistance to adults struggling during the pandemic, these programs play an important role for families with children as well. For low-income children during the pandemic, many are reliant on school meal programs to provide a substantial percentage of daily energy intake [11]. Adams et al. reported parents choosing to cut or skip meals in fear that they wouldn’t have enough to feed their children over the course of the pandemic. Among positive findings about the programs obtained from this study, one participant noted that the program not only lessened the burden of feeding her children nutritious food but also provided healthy ideas for feeding her children. The majority of study participants reported that they felt a sense of belonging in the community, enjoyed, and felt satisfied with the meals they had been receiving from their site. Information provided on how these programs may be used for nutrition education highlights an often-overlooked benefit that deserves further evaluation. Findings from this research highlight the potential positive impacts these meal assistance programs have on the nutritional status and dietary intake of marginalized populations by providing both increased access to nutritious food and guidance on healthier meal choices.

The unique approach of the meal distribution program used in this study not only targets FI in the short term but focuses on solutions to FI across the entire food system. By employing furloughed hospitality workers to prepare meals for the community, this program has a greater reach in addition to preventing FI amongst the furloughed workers by providing them with a wage as well as meal assistance. Paying restaurants and caterers to provide meals also increases the number of meals and distribution sites available to the community, while bolstering the local economy by preventing these entities from closing. Finally, through local procurement efforts for ingredients for the meals, both by the organization and the restaurants and caterers, this approach both prevents food waste and pays farmers for their products. Through this multi-prong approach, many solutions for FI are being addressed: for individuals in the community, for furloughed hospitality workers, for restaurants and caterers and their employees, and finally for farmers who provide food for the meals. This collaboration utilizes the skills and infrastructure that already exists in the community and allows for a rapid response in a disaster or pandemic. It also goes beyond just providing meals by being a means for employment if shutdowns occur, and becomes a solution to FI that can be easily be replicated across the country in any town or city with restaurants and kitchens. If we hope to target the root causes of FI, solutions to employment and wages are as much of a priority as access to food.

To be of service to the exponentially higher percentage of individuals needing assistance during the pandemic, many food assistance programs have more than doubled their typical output or have shifted their operation entirely [8]. According to the Food and Nutrition Services of the USDA, states are getting clearance to make programs more flexible and contingent to better serve its program participants without having to receive approval from the USDA. Fifteen nutrition programs are being granted these flexibilities [31]. As the pandemic will continue to create waves of issues through the health, finance, and food system, continued support is needed not only for the individuals requiring these programs, but for the programs themselves. Many individuals in the nation, who may never have needed these types of services before, have now increased the demand for, and requirement of, continuation of these efforts.

Among those in a disadvantaged SES group prior to COVID-19, the elevation of social and economic concerns during the pandemic has resulted in the inability to maintain a healthy life with adequate resources. Program importance data from our cohort identified the overarching themes of mental wellbeing as well as improved access and resources that the program had provided to recipients at our meal distribution sites during the pandemic. The importance of these services emerged through direct quote feedback from recipients mentioning the value and necessity of these meals to themselves and family. These data further underscore the continued need of these programs throughout the remainder of the COVID-19 pandemic and through its lasting, unknown, effects.

### Limitations

The current study is not without limitations due to its cross-sectional and self-reported nature. As the study was completed virtually due to research constraints during COVID-19, the self-reported nature of measures and results should be taken into consideration. Pre-COVID perceived stress levels were not measured before the COVID-19 pandemic, but individuals were asked to recall their memories regarding PSS-10 statements. Asking participants to recall stress can induce bias into the results and interpretation. Study participants were recruited through convenience sampling at the meal distribution site. As this study took place during COVID-19 while in-person research was restricted, convenience sampling by site staff was the only viable option while researchers were unable to be in-person. As our population was sourced via convenience sampling, generalizability to all meal assistance recipients should be cautioned for self-selection bias among those choosing to complete the survey. The study was additionally only in English which may have limited completion to those who were English-literate. Due to these limitations, generalizability to wider populations should be cautioned. Future studies should seek intentional sampling methods to gather more in-depth information with regard to COVID-19 impacts on food accessibility and the utilization of meal assistance programming in the community.

Despite its limitations, this study is one of the first aimed at understanding the population, or change in population, of meal service users throughout the COVID-19 pandemic and how the services are impacting their life and well-being. This initial study lends further insight into the community’s food needs, the importance of these meal assistance programs, particularly for those who have had an increased need since the COVID-19 pandemic, and areas for continued support.

## 5. Conclusions

Research that provides an analysis of the needs of a population along with an evaluation of the impact of meal assistance programs is critical for securing future funding and new partnerships for these organizations. Findings on meal assistance programs from this study will play a critical role when advocating for public policies that ensure the survival of these services as the pandemic continues. These food assistance programs remain an important factor in the health and wellbeing of millions of American families. More support on such public policies and research can provide the best practices for holistically preventing and/or addressing FI by going beyond the provision of food or meals and targeting health and wellbeing of emergency food assistance recipients not only during but also beyond the pandemic in the future.

## Figures and Tables

**Table 1:** Descriptive Statistics by Recipient and Non-Recipient Status.

Variable	Group	*N* (Mean ± SD/Percent/Range)
		Recipients (*n* = 92)
Sex	Male	26 (28.3%)
	Female	66 (71.7%)
Age (years)		43.5 ± 15.0
Race	White	34 (37.4%)
	Black	49 (52.7%)
	Other including bi-racial	9 (9.9%)
Child under 18 years old in household	Yes	39 (45.9%)
	No	46 (54.1%)
Number of children under 18 years old in household		2.3 ± 1.0 (1–5)
Income (USD)	Under 30,000	48 (52.2%)
	Above 30,000–Under 75,000	30 (32.2%)
	Above 75,000–Under 100,000	9 (10.0%)
	Above 100,000–150,000+	5 (5.6%)
Food Insecurity Status	Food Secure	5 (5.4%)
	Food Insecure	87 (94.5%)
Employment	Employed Pre-COVID	64 (70.3%)
	Employed Since-COVID	50 (56.8%)
Health Conditions	Type II Diabetes Mellitus	19 (22.9%)
	High Cholesterol	28 (34.1%)
	High Blood Pressure	37 (44.6%)
	Overweight	45 (52.9%)
	Obesity	29 (34.9%)
	Cancer	9 (11.0%)
	Depression	36 (43.4%)
	Anxiety	44 (53.0%)
Days consuming 4.5 servings of fruit & vegetables per week		3.4 ± 1.8
Missing Food Groups	Fruit	43 (46.7%)
	Vegetables	38 (41.3%)
	Dairy	31 (33.7%)
	Protein	25 (27.2%)
	Grains	20 (21.7%)
PSS-10	Pre-COVID	15.3 ± 5.3
	Post-COVID	22.7 ± 6.8

**Table 2:** Meal Recipient Raw Data Quote Examples with Main and Sub Themes.

Main Theme	Subtheme	Quote
Changed Habits	Healthy Eating	“Helped me have some nutritious food when I wouldn’t have had any”
Mental Wellbeing	Mental Health	“It has stretched my budget and allowed me to not have to move. I feel so much better than when I wasn’t receiving the extra help. I was anxious and overwhelmed.”
Provided Resources	Access	“This meal program has really [helped] my family and I eat meals every day. This pandemic was tough on us financially and being able to provide even a decent meal during these times was not always easy, but thanks to the help of these programs we managed to make it through and continuing to do so.”
	Financial	“They [kids] attend schools that provide free breakfast and lunch, and I did not budget for this during the pandemic so this has been a huge financial blessing.”
	Family and/or Community	“It has fed me and my grandkids We also help our neighbors with these meals. No job no food...”
Other	Other	“Seeing people I know, who need to satisfy their demands, who actually make it to some of the [meal distribution] locations, reflects its necessity.”

Data in Table 2 from Meal Program Recipients who have utilized the meal service.

## Data Availability

Data is available from the PI upon request.

## References

[1] Bureau of Labor Statistics Current Unemployment Rates for States and Historical Highs/Lows. https://www.bls.gov/web/laus/lauhsthl.html.

[2] Campbell E. (2019). Poverty Speaks: Making Tough Choices-The Center for Community Solutions.

[3] Despard M., Grinstein-Weiss M., Chun Y., Roll S. (2020). COVID-19 Job and Income Loss Leading to More Hunger and Financial Hardship.

[4] Karpman M., Zuckerman S., Gonzalez D., Kenney G.M. The COVID-19 Pandemic Is Straining Families’ Abilities to Afford Basic Needs. https://www.urban.org/research/publication/covid-19-pandemic-straining-families-abilities-afford-basic-needs?utm_source=SNEB+Members&utm_campaign=78869c6765-EMAIL_CAMPAIGN_2017_08_25_COPY_01&utm_medium=email&utm_term=0_5640af03cf-78869c6765-709893437.

[5] Coleman-Jensen A., Rabbitt M., Gregory C., Singh A. (2019). Household Food Security in the United States in 2018. ERR-270.

[6] Economic Research Service USDA Survey Tools. https://www.ers.usda.gov/topics/food-nutrition-assistance/food-security-in-the-us/survey-tools/.

[7] Hager E.R., Quigg A.M., Black M.M., Coleman S.M., Heeren T., Rose-Jacobs R., Cook J.T., de Cuba S.A.E., Casey P.H., Chilton M. (2010). Development and validity of a 2-item screen to identify families at risk for food insecurity. Pediatrics.

[8] Feeding America (2019). Map the Meal Gap.

[9] Gundersen C., Hake M., Dewey A., Engelhard E. (2020). Food insecurity during COVID-19. Appl. Econ. Perspect. Policy.

[10] Adams E.L., Caccavale L.J., Smith D., Bean M.K. (2020). Food insecurity, the home food environment, and parent feeding practices in the era of COVID-19. Obesity.

[11] Bauer L. (2020). The COVID-19 Crisis Has Already Left Too Many Children Hungry in America.

[12] Morales M.E., Berkowitz S.A. (2016). The relationship between food insecurity, dietary patterns, and obesity. Curr. Nutr. Rep..

[13] Laraia B.A. (2013). Food insecurity and chronic disease. Adv. Nutr..

[14] Seligman H.K., Schillinger D. (2010). Hunger and socioeconomic disparities in chronic disease. N. Engl. J. Med..

[15] Jih J., Stijacic-Cenzer I., Seligman H.K., Boscardin W.J., Nguyen T.T., Ritchie C.S. (2018). Chronic disease burden predicts food insecurity among older adults. Public Health Nutr..

[16] Nagata J.M., Palar K., Gooding H.C., Garber A.K., Bibbins-Domingo K., Weiser S.D. (2019). Food insecurity and chronic disease in US young adults: Findings from the National Longitudinal Study of Adolescent to Adult Health. J. Gen. Intern. Med..

[17] Seligman H.K., Laraia B.A., Kushel M.B. (2010). Food insecurity is associated with chronic disease among low-income NHANES participants. J. Nutr..

[18] Weaver L.J., Fasel C.B. (2018). A systematic review of the literature on the relationships between chronic diseases and food insecurity. Food Nutr. Sci..

[19] Gundersen C.Z.J. (2015). Food Insecurity And Health Outcomes. Health Aff..

[20] America F. How Feeding America Turns $1 into at Least 10 Meals. https://www.feedingamerica.org/ways-to-give/faq/about-our-claims.

[21] Morello P. (2020). 4 Stats You Should Know about Food Banks and COVID-19.

[22] Agricultural Marketing Service, U.S. Department of Agriculture USDA Farmers to Families Food Boxes. https://www.ams.usda.gov/selling-food-to-usda/farmers-to-families-food-box.

[23] Benefits.gov Kentucky Food Benefits/EBT. https://www.benefits.gov/benefit/1213.

[24] FoodChain (2020). End of Year Impact Numbers.

[25] FoodChain (2021). Nourish by the Numbers.

[26] Straub S. New Map Fights Food Insecurity in Lexington. *Lexington-Fayette Urban County Government News* 2020. https://www.lexingtonky.gov/news/11-24-2020/new-map-fights-food-insecurity-lexington.

[27] Qualtrics Inc. www.qualtrics.com.

[28] Horowitz J.M., Minkin A.B.R. A Year Into the Pandemic, Long-Term Financial Impact Weighs Heavily on Many Americans. https://www.pewresearch.org/social-trends/2021/03/05/a-year-into-the-pandemic-long-term-financial-impact-weighs-heavily-on-many-americans/.

[29] Ettman C.K., Abdalla S.M., Cohen G.H., Sampson L., Vivier P.M., Galea S. (2020). Prevalence of depression symptoms in US adults before and during the COVID-19 pandemic. JAMA Netw. Open.

[30] Wang P., Fang J., Gao Z., Zhang C., Xie S. (2016). Higher intake of fruits, vegetables or their fiber reduces the risk of type 2 diabetes: A meta-analysis. J. Diabetes Investig..

[31] Food and Nutrition Services FNS Responds to COVID-19. https://www.fns.usda.gov/coronavirus.

